# Molecular Peptide Grafting as a Tool to Create Novel Protein Therapeutics

**DOI:** 10.3390/molecules28052383

**Published:** 2023-03-05

**Authors:** Anton A. Komar

**Affiliations:** 1 Center for Gene Regulation in Health and Disease, Department of Biological, Geological and Environmental Sciences, Cleveland State University, 2121 Euclid Avenue, Cleveland, OH 44115, USA; a.komar@csuohio.edu; Tel.: +1-216-687-2516; 2 Department of Biochemistry and Center for RNA Science and Therapeutics, School of Medicine, Case Western Reserve University, Cleveland, OH 44106, USA; 3 Genomic Medicine Institute, Lerner Research Institute, Cleveland Clinic, Cleveland, OH 44195, USA

**Keywords:** peptide therapeutics, molecular peptide grafting, hydrogen bond surrogates, molecular staples, cyclic peptides, cyclotides, GST, TNF-α, Mdm2, p53

## Abstract

The study of peptides (synthetic or corresponding to discrete regions of proteins) has facilitated the understanding of protein structure–activity relationships. Short peptides can also be used as powerful therapeutic agents. However, the functional activity of many short peptides is usually substantially lower than that of their parental proteins. This is (as a rule) due to their diminished structural organization, stability, and solubility often leading to an enhanced propensity for aggregation. Several approaches have emerged to overcome these limitations, which are aimed at imposing structural constraints into the backbone and/or sidechains of the therapeutic peptides (such as molecular stapling, peptide backbone circularization and molecular grafting), therefore enforcing their biologically active conformation and thus improving their solubility, stability, and functional activity. This review provides a short summary of approaches aimed at enhancing the biological activity of short functional peptides with a particular focus on the peptide grafting approach, whereby a functional peptide is inserted into a scaffold molecule. Intra-backbone insertions of short therapeutic peptides into scaffold proteins have been shown to enhance their activity and render them a more stable and biologically active conformation.

## 1. Introduction

**Figure 1:**
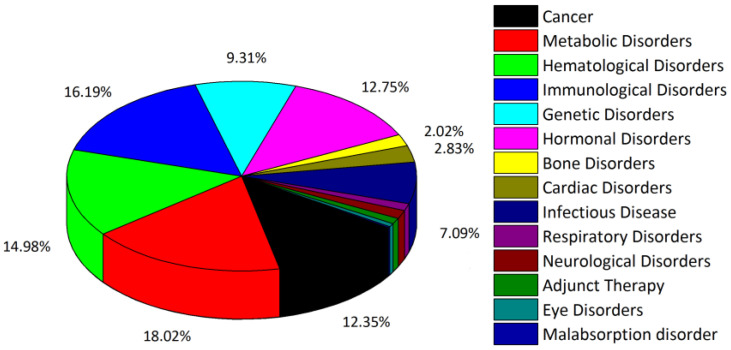
Disease/disorders targeted by therapeutic peptides and proteins according to the summary statistics of the THPdb database of FDA-approved therapeutic peptides and proteins [13,14]. The data were plotted using THPdb statistics page [14] information. Numerical values for under-represented disorders (<2%), such as Respiratory Disorders (~1.21%), Neurological Disorders (~1.42%), Adjunct Therapy (~1.01%), Eye Disorders (~0.61%) and Malabsorption disorder (~0.20%) are omitted on the pie chart for the ease of reading.

Proteins are polymers of amino acids that are constructed from a number of structurally and/or functionally conserved modules that are as a rule genetically mobile and used repeatedly in the course of evolution [1,2,3]. As such, proteins are often described as assemblies of “Lego bricks”, referring to their modular nature [1,2,3]. These modules/bricks may be relatively large, constituting protein subdomains/domains of about 50–250 amino acids in length (it has been widely recognized that proteins are often divided into subdomains of ~50 amino acid residues in lengths and domains of ~100–250 amino acid residues in length [4,5,6]), or quite small and represented by short peptides composed of ~5–40 amino acids [2,7]. Importantly, such small peptides can yet possess selected biological activities of the full-length proteins from which they were derived [8,9,10,11,12]. At the same time, such short peptides would be lacking (potentially) unwanted activities associated with the rest of the protein and thus can have targeted clinical applications [8,9,10,11,12]. To date, over 80 peptide therapeutics have been approved in the United States and other countries in the world with over 500 being in development and/or entering clinical trials [8,9,10,11,12]. Peptides have been investigated across a broad therapeutic spectrum, revealing their utility across a wide range of disease indications [8,9,10,11,12]. The so-called THPdb, a database of FDA-approved therapeutic peptides and proteins, currently lists 852 entries and provides comprehensive information on 239 therapeutic peptides/polypeptides (already on the market and/or in various phases of clinical trials) [13]. It also provides information on the 380 drug variants of these therapeutic peptides and proteins [13]. The database provides a detailed overview of peptide and protein properties including their sequences/composition, chemical properties, structure (if available), disease area, mode of activity, physical appearance, category or pharmacological class, pharmacodynamics, routes of administration, associated toxicity (if exists), and so forth. Major diseases targeted by these peptides and proteins include cancer, metabolic disorders, hematological disorders, immunological, and hormonal disorders [13] (Figure 1).

About 23% of all the protein and peptide therapeutics listed in the THPdb database are represented by polypeptides shorter than 60 amino acid residues in length [13,15], and of these about a half (11%) is represented by peptides shorter than 30 amino acids in length [15]. It must be noted, however, that the functional activity of many short peptides (especially the ones shorter than 15 amino acids in length) was found to be usually substantially lower than that of their parental proteins [16,17,18,19,20]. In general, this is due to their diminished structural organization, solubility, and stability often leading to an enhanced propensity for aggregation [8,9,10,11,12,16,17,18,19,20,21,22]. Other limitations, such as short plasma half-life and overall poor oral bioavailability also diminished initial enthusiasm for peptide therapeutics [8,9,10,11,12]. Thus, a number of more nuanced approaches have emerged aimed at enhancing the solubility, stability, and functional activity of peptide therapeutics [8,9,10,11,12,16,17,18,19,20,21,22,23,24,25,26,27,28,29,30,31,32]. In particular, N- or C-end-terminal peptide-fusions has emerged as a potential solution to alter and enhance the properties of peptide therapeutics [23,24,25]. Nonetheless, fusion peptides may remain flexible and unstable in a solution [16,18,19,20]. Fusion proteins containing functional peptides on their N- or C-termini also often do not allow sufficient structural constraints for the enhancement of activity and/or solubility. As alternative strategies for the enhancement of peptide activity, short peptides can be stabilized by intramolecular covalent linkages (staples) that reinforce their structure [30,31,32] and/or inserted into the backbones of biologically inert (relevant to the targeted process) proteins that otherwise possess other desired properties of, for example, high solubility, stability, and ease of purification [26,27,28,29]. These approaches also allow imposing the necessary structural constraints, required for peptide function as therapeutics.

This review provides a short summary of approaches aimed at enhancing the biological activity of short functional peptides with a particular focus on the peptide grafting approach [26,27,28,29], whereby a functional peptide is inserted into a scaffold molecule. Intra-backbone insertions of such small peptides into scaffold proteins have been shown to enhance the biological activity of the short therapeutic peptides and render them a more stable and biologically active conformation.

## 2. Functional Peptides

The study of peptides, either synthetic and/or corresponding to discrete regions of proteins, has facilitated our understanding of protein structure–activity relationships [1,2,7]. A peptide is a compound consisting of at least two amino acids linked in a chain; however, the upper amino acid chain limit for a peptide is not well defined and frequently is established arbitrarily. The National Human Genome Research Institute (NHGRI) defines a peptide as a “short chain of amino acids (typically 2 to 50) [33]”. According to the NHGRI, a longer chain of linked amino acids (51 or more) would be called a polypeptide and/or a protein. Nevertheless, very often (in the literature) even chains longer than 51 amino acids are called peptides. Yet, the basic distinguishing factor is the size (2–50 amino acids for a peptide) and often the structure, as peptides tend to possess less well defined structures (in solution).

**Figure 2:**
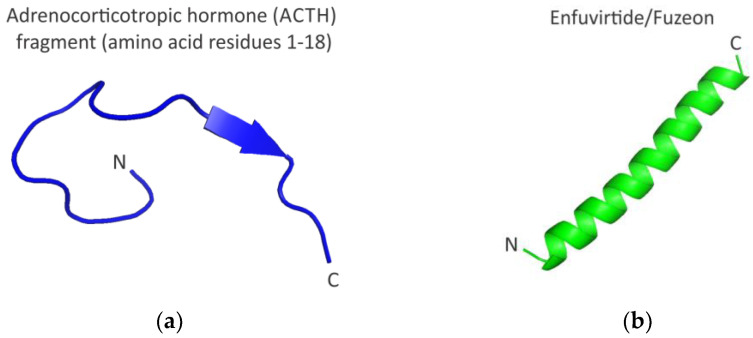
Structures of an adrenocorticotropic hormone (ACTH) fragment and Enfuvirtide/Fuzeon peptides. (**a**) The structure of the ACTH fragment (amino acids 1–18) is visualized using PDB ID 8GY7. This PDB entry presents a Cryo-EM structure of the ACTH-bound melanocortin-2 receptor in complex with the melanocortin receptor accessory protein MRAP1 and melanocortin receptor 2 (MC2R). Note that the structure of the full-length ACTH in solution has not been solved to date. It is believed that ACTH is largely unstructured in solution and undergoes conformational changes upon binding to the MC2R [38]; (**b**) the structure of Enfuvirtide/Fuzeon is visualized using PDB ID 5ZCX.

From the clinical perspective, peptides represent a unique class of pharmaceutical compounds, as they are distinct from both small molecules and proteins, but provide a rather unique opportunity for therapeutic intervention that targets and/or closely mimics natural pathways [8,9,10,11,12]. The utilization of peptides as therapeutics has dramatically evolved over time [8,9,10,11,12] from the use of peptides isolated from the natural sources, such as bovine and/or porcine pituitary glands, from which a highly conserved 39 amino acid adrenocorticotropic hormone (ACTH; corticotrophin [34] (Figure 2a) was originally isolated, to the use of recombinant and/or synthetic peptides, such as 36-amino acid anti-HIV-1 peptide Enfuvirtide/Fuzeon [35,36,37] (Figure 2b). One has to note, however, that the early peptide therapeutics, like ACTH (that was/is used to treat certain endocrine disorders [34]), represented natural products derived in cells from the precursor polypeptides (in case of ACTH-256 amino acid precursor pre-pro-opiomelanocortin protein [34]).

The new genomic and genetic engineering era in molecular biology has allowed for the identification, expression/synthesis, isolation, and molecular characterization of the protein-integral peptide fragments that do not exist in nature separately from the underlying proteins, but were found to have distinct biological activities. It should be noted in this regard that Enfuvirtide/Fuzeon (an inhibitor of HIV-1 entry into CD4+ cells, which prevents virus–host cell membrane fusion) was rationally derived from the HIV-1 envelope glycoprotein gp41 (gp41 region comprising amino acid residues 638–673) [35,36,37]. Similarly, for example, ~20–25-amino acid peptides that were shown to be capable of inhibiting T cell adhesion and function, were derived from the large (>500 amino acids each) sequences of the two cell adhesion counter receptors, leukocyte function-associated antigen (LFA)-1 and intercellular adhesion molecule (ICAM)-1 [39]. It should be noted that blocking the T-cell adhesion is in important therapeutic strategy as it can suppress the progression of autoimmune diseases and prevent allograph rejection [40].

It has been quickly realized that specific regions of the relatively “long” ~25–40 amino acid peptides that are responsible for their activity (e.g., inhibitory activity like in case of, for example, LFA-1 and ICAM-1) could be even much smaller and represented by substantially shorter fragments of about 10–14 amino acid residues in length [41]. However, it has been also found that such shorter peptides may be substantially less active in solution, as structural studies indicated that they may lack the structural elements required to emulate the biological activity of the native protein [8,9,10,11,12,16]. These results suggested that the desired functional activity in short peptides, besides the presence of specific amino acids, may require the existence of specific and stable structural features.

## 3. Imposing Conformational Constraints in Short Peptides

Functional peptides are frequently composed of α-helices or a single helix, which play import roles in mediating protein–protein interactions [42,43]. However, such short helical peptides, which within a protein adopt a defined conformation, typically do not fold into a stable helical structure in solution [42,43,44]. Therefore, studies have been initiated that aim at enhancing the biological activity of short peptides via enforcing their desired structural features. Numerous approaches have been developed [16,17,18,19,20,21,22,23,24,25,26,27,28,29,30,31,32,45,46,47,48,49,50]. I will mention just a few to reveal the tip of iceberg and introduce the readers to some of the studies conducted in the field with a particular focus on peptide transplantation or peptide-grafting approach [26,27,28,29].

### 3.1. Hydrogen Bond Surrogates and Staples

**Figure 3:**
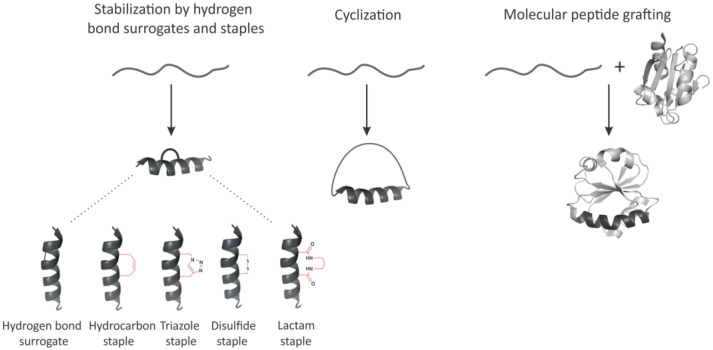
Representative stabilization methods for short peptides. Peptide backbone stabilization can be achieved via a hydrogen bond surrogate strategy and/or introduction of staples (**left**), peptide cyclization (**middle**) and/or grafting onto a desired scaffold (**right**).

Given the importance of the α-helical peptides as therapeutic agents, several key strategies to stabilize this conformation in peptides and/or enhance/mimic it with non-natural scaffolds have evolved over time [30,31,32,45,46,47,48,49,50]. One such strategy involves the preparation of artificial α-helices containing the replacement of one of the main chain hydrogen bonds with a covalent linkage [45] (Figure 3). The main chain hydrogen bond surrogate strategy with a placement of the cross-link on the inside of the helix does not block solvent-exposed molecular recognition surfaces of the peptide and thus was considered to be superior to the other α-helix stabilization methods that have relied on side-chain constraints, which may block solvent-exposed surfaces of the target α-helices or diminish important side-chain functionalities [45]. Another, widely used, so-called “staple” approach [30,31,32,47,48,49,50] (Figure 3), introduced the use of, for example, α,α-disubstituted non-natural amino acids containing olefin-bearing tethers to generate an all-hydrocarbon “staple” in short peptides by ruthenium-catalyzed olefin metathesis. A variety of other modifications including but not limited to triazole staples synthesized from alkenyl and azido side chain residues, disulfide bridges and lactam bridges, have been introduced [50] (Figure 3). However, some of these approaches may yet require sophisticated and costly chemical synthesis as well as purification steps making them in many instances less attractive.

### 3.2. Peptide Backbone Circularization

An additional method which generated substantial interest among researchers and biotechnology industry is peptide circularization (Figure 3). Circularization (or cyclization) can be achieved via a number of strategies, such as N- and C-terminal end ligation or head-to-sidechain, sidechain-to-tail, or sidechain-to-sidechain ligation [51,52]. The increasing number of chemistries compatible with in vivo systems attracted special attention to cyclization and undoubtedly accelerated drug discovery in the field of functional short peptides. In the last few decades many simple and affordable approaches such as disulfide cyclization have attracted particular attention [50,51,52]. The specific distances of disulfide bonds connected to cysteines was found to be best suited especially for stabilizing short α-helical (i, i+7) peptides. Cyclization was indeed shown to promote a higher level of structural organization in such short peptides [51,52] and enhance their biological activity, as has been demonstrated using, for example, the short LFA-1 and ICAM-1 peptides mentioned above [16]. However, many challenges associated with the use of, for example, disulfide cyclization still require further attention. Disulfides, for example, are well known to be inherently unstable in a reducing environment and to improve their stability, additional applications have been developed, where disulfide groups have been substituted with, for example, the lactam, thioether, selenium, triazole, or dicarba analogues also mentioned above [50,51]. Given these and other considerations, it has been suggested that cyclization approaches would yet require significant and non-trivial chemical synthesis steps [50,51,52].

### 3.3. Molecular Peptide Grafting

Molecular grafting is an emerging strategy for the engineering of molecular scaffolds with novel functions which has attracted increasing attention in recent years as a plausible tool for the development of next-generation protein therapeutics. Grafting involves taking a bioactive protein fragment and transferring it onto a desired scaffold, therefore creating a chimera, which can possess unique properties that would in many instances be more than a sum of two [26,27,28,29] (Figure 3). The insert can be as big as the entire protein domain or as small as a peptide. Despite its attractive nature, molecular grafting studies thus far have focused mainly on demonstrating the utility and applicability of the approach rather than developing robust strategies leading to the broad application of the approach [26,27,28,29]. It should be noted that obviously not every protein would tolerate insertions (and not in every region) and identifying scaffolds more suitable for grafting and formalizing such features represents a largely unmet need in the field [26,27,28,29].

I will review below just a few representative examples (including our own studies) illustrating the approach with a focus on insertions of short peptides into larger protein scaffolds and briefly discuss some of the advantages and disadvantages of this approach.

#### 3.3.1. Chimeric Glutathione S-Transferases (GSTs) Containing Inserts of Kininogen Peptides as Cellular Anti-Proliferating Agents

**Figure 4:**
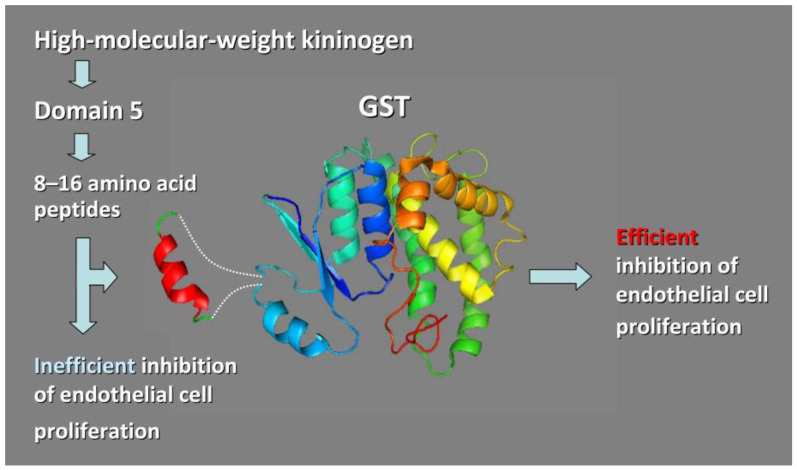
Grafting of short human high molecular weight kininogen (HK) peptides onto Glutathione S-Transferase (GST) backbone enhances HK peptide’s ability to inhibit endothelial cell proliferation [46]. GST on its own does not inhibit cell proliferation.

About a decade ago we presented the design and functional characterization of engineered GST proteins carrying 8–16-amino acid peptide inserts [53] (Figure 4). These peptides were derived from a sequence within domain 5 (D5) of human high molecular weight kininogen (HK) [17,54,55,56,57,58]. HK domain 5 contains endothelial cell-binding sites and inhibits angiogenesis through its ability to cause apoptosis of proliferating endothelial cells as well as to inhibit endothelial cell proliferation and migration [54,55,56,57,58]. Short histidine-glycine-lysine (HGK) peptides derived from this domain were also shown to block tumor metastasis, therefore representing an attractive target for the design of antitumor peptide therapeutics/drugs [17,54,55,56,57,58]. However, the short HGK peptides were found to be much less active than the parental domain/protein, especially when used at lower concentrations [53]. To enhance their activity, we grafted these peptides into the backbone of GST [53]. We engineered eight chimeric GST proteins (termed GSHKTs) in which HGK peptides ranging in size from 8- to 16-amino acids were inserted into *Schistosoma japonicum* GST (between Gly-49 and Leu-50) [53]. We chose to use GST as a scaffold because the protein is well studied [59] and is widely used for the affinity purification of fusion proteins expressed in *Escherichia coli*, and importantly, because it does not inhibit cell proliferation [17,58]. We characterized the grafted proteins in terms of their biological activity (by assessing their ability to inhibit human umbilical vein endothelial cell proliferation in a dose-dependent manner) and also thermostability (using differential scanning calorimetry). We also solved the crystal structures of GSHKT10 and GSHKT13 chimeras (harboring HK peptides of 10 and 13 amino acid residues in length, respectively) [53]. As such, we were able to demonstrate that constraining the flexibility of HGK peptides through insertion into the GST backbone, substantially enhances their biological activity, increasing it up to ~100-fold compared with the free peptides. Importantly, grafting did not perturb the overall GST structure, thus allowing the efficient affinity purification of the chimeric proteins [17].

To our knowledge, this was the first report demonstrating the use of GST as a grafting scaffold. It should be noted in this regard that the production of the HGK motif containing peptides expressed as fusions with various recombinant proteins did not enhance their biological activity [17,54,55,56,57,58], thus revealing the obvious advantage of the grafting approach.

We found, however, that the chimeric GSHKT proteins were thermodynamically less stable than isolated GST (with a ~8–10 °C lower phase transition temperatures compared to GST [53]), revealing a well-known and common trade off in molecular grafting studies. Nevertheless, all the GSHKT chimeras were soluble upon high expression *E. coli*, therefore addressing major demands for biologically active peptides (to function as therapeutic agents), i.e., efficient production, high solubility, and retention and/or enhancement of biological activity. It should be noted that in our studies, we used GST from a helminth *S. japonicum* and in humans this scaffold may trigger an unwanted immune response. However, GSTs are structurally conserved molecules [59] and human GSTs (belonging, for example, to mu or pi classes [59]) can be used as scaffolds instead.

#### 3.3.2. TNF-α Epitope-Scaffold Immunogen

Tumor necrosis factor (TNF)-α is pro-inflammatory cytokine which plays important roles in many physiological and pathological processes [60]. Inhibition of TNF-α activity with, for example, monoclonal antibodies as well as a receptor-immunoglobulin fusion protein has been considered an important strategy for improving clinical outcomes in patients with unresolved inflammation and associated autoimmune disorders, such as rheumatoid arthritis [61,62,63,64,65,66]. Current TNF-α biological inhibitors are, however, limited in practice due to associated side effects [64,65]. Immunization against TNF-α has been intensively investigated as an alternative approach to treat unresolved inflammation and address certain limits of the available biologics. Since TNF-α is a very potent cytokine, a whole molecule immunogen cannot be used and needs to be inactivated before use (either by chemical treatment [67] or site-directed mutagenesis [68]). Such vaccine strategies were, however, not successful in human trials. Therefore, a peptide epitope-based vaccine design approach was considered as a preferred choice [69]. However, only weak or transient antibody responses were observed with linear peptides and peptide conjugates [61,62,63,64,65,66,69]. At the same time, a cyclic TNF-α epitope peptide (comprising TNF-α amino acids 80–96) was shown to elicit a much stronger neutralizing antibody response, indicating the necessity of conformational constraints [69]. Following this consideration, Zhang and colleagues recently developed an epitope-scaffold immunogen against TNF-α, in which the conformation of the short peptide derived from TNF-α (amino acids 80–97) was stabilized by insertion into a scaffold molecule, a transmembrane domain of diphtheria toxin (DTT) [70]. DTT is safe as an epitope carrier because it represents a catalytically inactive fragment of the diphtheria toxin [70]. The authors used the “cut and paste and cut and replace” strategy to construct seven chimeric proteins, whereby various numbers of amino acid residues at position 89–96 on the diphtheria toxin T-domain were replaced by the 18-amino-acid-long TNF-α epitope [70]. The immunogenicity of the grafted chimeras against native TNF-α in mice as well as the therapeutic efficacy in a collagen-induced arthritis mouse model were then investigated. Vaccination with a grafted scaffold was shown to inhibit arthritis development in the collagen-induced arthritis mouse model [70]. However, similarly to our and other studies, the thermostability of all grafted proteins appeared to be lower than that of DTT (on average ~3 °C to ~9 °C lower) [70]. Nevertheless, all of the chimeras were highly soluble at a concentration greater than 10 mg/mL in Phosphate-buffered saline (PBS) pH 7.4, at 25 °C. These results suggested that TNF-α epitope DTT-based scaffold vaccine may represent a promising strategy for the prevention and treatment of autoimmune diseases. It remains to be established, however, whether or not an immune response towards DTT itself may complicate the use of this scaffold.

#### 3.3.3. Grafting Short hDM2/Mdm2 Hydrophobic Epitopes as a Tool to Inhibit Protein–Protein Interactions Critical for Cancer Development and Progression

Hydrophobic interactions were suggested to be critical for establishing protein–protein interactions and provide the bulk of binding energy [71,72,73]. Thus, approaches that graft such critical hydrophobic epitopes onto other structurally constrained scaffolds were developed [74,75,76,77,78,79,80]. As above, the inhibition abilities/activities of these peptides were studied in vitro (in test tube) and/or ex vivo/in vivo in cellular and model organisms. Many such studies [74,75,76,77,78,79,80] were focused on human double-minute 2 oncoprotein (hDM2; also known as Mdm2) a principal cellular antagonist of the major tumor-suppressor protein p53 [81]. Elevated hDM2 levels were found in many solid tumors that express wild-type p53 and were associated with poor prognosis and disease outcomes [81]. Thus, there was a considerable interest in hDM2/Mdm2 ligands that block hDM2–p53 interaction and, as a consequence, upregulate p53 activity. The high-resolution structure of the hDM2 complexed with p53 activation domain (AD) has revealed that a recognition epitope is composed primarily of a short α-helix [82]. As such, several scaffolds have been designed to display the p53AD epitope [74,75,76,77,78,79,80,83]. These included, for example, (relatively) large scaffold proteins (such as thioredoxin [83]), small polypeptides, like avian pancreatic polypeptide [83] or scorpion toxin [78], as well short constraint peptide scaffolds [76,77]. The affinity and inhibitory activity of such scaffolds have been further investigated and it has been found that grafting does enforce higher levels of the α-helical structure that is critical for the functional activity of the peptide insert, resulting in the higher affinity and activity of the grafted scaffold [74,75,76,77,78,79,80,83]. Expression of such scaffolds was shown to disrupt Mdm2–p53 interactions and activate p53-dependent transcription [83]. These data provided further evidence that protein grafting, in combination with functional selection, provides a powerful tool to block disease relevant protein–protein interactions. However, the proper choice of a scaffold appeared to be critically important. It was found that certain chimeras may not inhibit the desired interaction in the cellular systems and may reveal off-target activities. In a recent study, where residues critical for Mdm2–p53 interaction were grafted onto the framework of a cell-penetrating peptide (termed CADY2)), the grafted scaffold was found to not inhibit the Mdm2–p53 interaction [80]. Pull-down experiments followed by proteomic analysis led to the identification of several off-target protein candidates [80]. This result suggests that the grafting strategy should be used with great caution and a grafted protein therapeutic should undergo extensive cellular and model organism(s) studies before entering the clinic.

#### 3.3.4. Cyclotide Scaffolds Targeting Biomolecular Interactions

**Figure 5:**
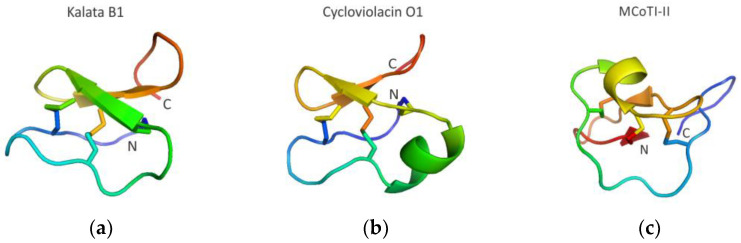
Structure of the main cyclotide subfamilies. (**a**) Structure of the Möbius, kalata B1, cyclotide PDB ID 1NB1); (**b**) structure of the bracelet, cycloviolacin O1 cyclotide, PDB ID 1NBJ; (**c**) structure of the trypsin inhibitor, MCoTI-II cyclotide, PDB ID 1IB9. Disulfide bridges are shown as sticks.

Among different scaffolds, the so-called cyclotide family of peptides has attracted special attention [84,85]. Cyclic peptides have been found in all domains of life [84,85], however, short (~30–40 amino acids long), cysteine-rich plant-derived backbone-cyclized peptides have received increased attention for developing novel protein therapeutics [27,29,76,86]. Cyclotides have six Cys residues that form a Cys-knotted structure (Figure 5) possessing enhanced stability to thermal denaturation and proteolytic degradation [76,86]. Importantly, these knotted peptides possess remarkable ability to accommodate large insertions in their loop regions, tolerating peptides ranging in size from 14 to up to 25 amino acid residues in length [76,86].

Naturally occurring cyclotides have been classified into three main subfamilies, the so-called Möbius (represented, for example, by kalata B1 cyclotide (Figure 5a)), bracelet (represented, for example, by cycloviolacin O1 cyclotide (Figure 5b)), and trypsin inhibitor (represented, for example, by MCoTI-II cyclotide (Figure 5c)) cyclotide subfamilies [27,29]. The first cyclotide discovered in plants (extracted from the African plant *Oldenlandia affinis*), kalata B1 (Figure 5a), was originally used in traditional medicine as an effective uterotonic agent (used by indigenous people in central Africa to accelerate childbirth) and was given orally [87]. Natural cyclotides from various sources have been shown to possess a plethora of different activities, such as, for example, cytotoxic/anticancer, antibacterial, hemolytic, antifungal, immunosuppressive, protease-inhibitory, and anti-HIV [88].

In 2008, Gunasekera and colleagues designed a vascular endothelial growth factor (VEGF) antagonist by grafting the hexamer “RRKRRR” peptide (involved in VEGF−A antagonism) onto the kalata B1 scaffold [89]. A grafted chimera showed anti-VEGF activity in an in vitro assay [89]. The in vitro stability of the target epitope was found to be also markedly increased [89].

The seminal work of Julio Camarero and colleagues revealing that MCoTI-II cyclotide carrying hDM2/Mdm2 peptides can activate the p53 tumor suppressor pathway and block tumor growth in a human colorectal carcinoma xenograft mouse model [76] opened up a new chapter of research in the field as it relates to the anti-cancer activity of grafted cyclotides [27,29]. Cys-rich cyclic peptide scaffolds have also shown significant promise for the treatment of diabetes, multiple sclerosis, and infectious diseases [90].

Thus far, cyclotides belonging to Möbius (kalata B1) and trypsin inhibitor (MCoTI-II) subfamilies were the most frequently used cyclotide scaffolds in molecular grafting [90]. The stable macrocyclic structure of cyclotides appeared to be suitable for introducing a variety of therapeutic grafts, including both structured epitopes, such as helical regions (involved in protein–protein interactions), as well as less well-structured functional loop regions [27,29]. Importantly, in all the studied cases, the grafted peptides exhibited higher functional activity than the linear peptide counterparts and peptide-mimetics [90]. However, one of the main problems with protein-based grafted therapeutics (including grafted cyclotide scaffolds), i.e., their ability to trigger an unwanted immune response, is yet to be thoroughly investigated and resolved. The pre-licensure immunogenicity assessment of novel therapeutic peptides and proteins is the subject of many recent research and clinical trials [91].

## 4. Conclusions and Future Perspectives

Advances in molecular modeling, structural predictions, and genetic manipulations have revolutionized our ability to design novel proteins. These approaches have also allowed the development of peptide therapeutics derived from natural proteins—a major breakthrough that continues to advance the field of drug development. For peptides to function as pharmacologically active agents, efficient production, high solubility, and retention of biological activity are required. However, many isolated peptides have a diminished structural propensity and do not adopt the conformation required for their function. Therefore, several approaches have been developed to overcome this limitation. In particular, molecular grafting has emerged as a promising strategy for the engineering of next-generation peptide therapeutics. Molecular grafting involves the transplantation of one protein fragment into a scaffold protein to endow chimera with a new and improved function. This approach proved its applicability and paved the way to the development of novel protein therapeutics. However, the configurational and thermodynamic stability features of the grafted scaffold remain one of the concerns yet to be addressed. In addition, potential (unwanted) immunogenicity remains a subject of another constant concern. Therefore, the methods and approaches to identify and/or design scaffolds with high stability allowing accommodating grafts without substantial penalty both in terms of thermal stability, off target reactions, and immunogenicity will be the subject of future studies and undoubtedly add higher value and broader applicability to the novel generation of grafted protein therapeutics.

## Data Availability

Not applicable.
